# Effects of 6-Month Sitagliptin Treatment on Glucose and Lipid Metabolism, Blood Pressure, Body Weight and Renal Function in Type 2 Diabetic Patients: A Chart-Based Analysis

**DOI:** 10.4021/jocmr975w

**Published:** 2012-07-20

**Authors:** Hidekatsu Yanai, Hiroki Adachi, Hidetaka Hamasaki, Yoshinori Masui, Reo Yoshikawa, Sumie Moriyama, Shuichi Mishima, Akahito Sako

**Affiliations:** a Department of Internal Medicine, National Center for Global Health and Medicine, Kohnodai Hospital, Chiba 272-8516, Japan; b Clinical Research Center, National Center for Global Health and Medicine, Kohnodai Hospital, Chiba 272-8516, Japan

**Keywords:** Body weight, Chart-based analysis, Hemoglobin A1c, Sitagliptin, Urinary glucose

## Abstract

**Background:**

Sitagliptin is one of the dipeptidyl peptidase-4 (DPP-4) inhibitors which prevent the inactivation of incretins, increasing the endogenous active incretin levels. Incretins stimulate insulin secretion from pancreatic β-cells and inhibit glucagon secretion from pancreatic α-cells, which is favorable for the treatment of diabetes. Sitagliptin is released on December, 2009, in Japan. We retrospectively studied effects of 6-month-treatment with sitagliptin on glucose and lipid metabolism, blood pressure, body weight and renal function in patients with type 2 diabetes by a chart-based analysis.

**Methods:**

We retrospectively studied 220 type 2 diabetic patients who have taken sitagliptin for 6 months by a chart-based analysis. Subjects studied include patients treated with sitagliptin monotherapy, sitagliptin add-on therapy, and switching from glinide to sitagliptin. We selected patients who have both data before and after 6-month sitagliptin treatment and compared the data before the sitagliptin treatment with the data at 6 month after the sitagliptin treatment started. Body weight, blood pressure, plasma glucose, hemoglobin A1c (HbA1c), serum lipids, and estimated glomerular filtration rate in type 2 diabetic patients were measured almost at the same time points before and after 6-month-treatment with sitagliptin.

**Results:**

Body weight was significantly reduced after 6-month sitagliptin treatment by 0.8 kg. HbA1c levels were also significantly decreased after the sitagliptin treatment by 0.6%. We found a significant and negative correlation between change in body weight and body mass index at baseline. We also observed a significant and negative correlation between change in HbA1c and HbA1c levels at baseline. The number of patients who showed the absence of urinary glucose was significantly increased after the sitagliptin treatment.

## Introduction

Incretins such as the glucagon-like peptide-1 (GLP-1) and the glucose-dependent insulinotropic polypeptide (GIP) are released from the intestinal cells following meal ingestion [1-3]. The GLP-1 and GIP stimulate insulin secretion from pancreatic β-cells and the GLP-1 inhibits glucagon secretion from pancreatic α-cells, which reduces plasma glucose levels [1-3]. However, incretins are rapidly inactivated by the dipeptidyl peptidase-4 (DPP-4) after released from the intestinal cells [1, 3]. Sitagliptin is one of the DPP-4 inhibitors which prevent the inactivation of incretins, increasing the endogenous active incretin levels [1, 3]. Hypoglycemia is very rare (less than 3%) during treatment with sitagliptin as monotherapy or in combination with metformin or thiazolidinediones [3, 4-10]. Several studies demonstrated that sitagliptin do not increase body weight compared to thiazolidinediones, sulfonylurea and insulin [2, 6-12]. A low frequency of hypoglycemia and weight gain in patients treated with sitagliptin may be explained by incretin-mediated glucose-dependent insulin secretion.

We retrospectively studied effects of 6-month-treatment with sitagliptin on glucose and lipid metabolism, blood pressure, body weight and renal function in patients with type 2 diabetes by a chart-based analysis.

## Materials and Methods

### Subjects

We retrospectively studied 220 type 2 diabetic patients who had taken sitagliptin for 6 months by a chart-based analysis. Clinical and biochemical characteristics of subjects studied were shown in Table 1. Other oral antihyperglycemic agents which subjects had taken before the sitagliptin treatment were shown in Table 2. Subjects studied include patients treated with sitagliptin monotherapy, sitagliptin add-on therapy, and switching from glinide to sitagliptin. We always stopped glinide when we started to use sitagliptin because the co-administration of sitagliptin with glinide is not approved by the health insurance system in Japan.

**Table 1:** Clinical and Biochemical Characteristics of Subjects Studied

Number of subjects	220
Age (years old)	64.0 ± 14.0
Sex (male/female)	102/118
Body height (cm)	160.0 ± 8.9
Body weight (kg)	68.2 ± 15.8
Body mass index (kg/m^2^)	26.1 ± 5.3
Systolic blood pressure (mmHg)	126.7 ± 15.5
Diastolic blood pressure (mmHg)	69.8 ± 13.6
Plasma glucose (mg/dL)	185.5 ± 69.3
Hemoglobin A1c (%)	8.1 ± 1.3
Serum LDL-C (mg/dL)	105.0 ± 28.5
Serum TG (mg/dL)	176.7 ± 122.0
Serum HDL-C (mg/dL)	50.7 ± 14.6
e-GFR (mL/min./1.73m^2^)	77.4 ± 24.4

Presented values indicate mean ± S.D., e-GFR, estimated glomerular filtration rate; HDL, high-density lipoprotein; LDL-C, low-density lipoprotein-cholesterol; TG, triglyceride.

**Table 2:** Other Oral Hypoglycemic Agents Which Subjects had Taken Before the Treatment With Sitagliptin

No other drugs	15
Sulfonyl urea	80
Biguanide	122
Pioglitazone	84
α-glucosidase inhibitor	80
Glinide	26

### Methods

This study was approved by the Institutional Ethics Committee in National Center for Global Health and Medicine, Japan. We selected patients who have both data before and after 6-month sitagliptin treatment and compared the data before the sitagliptin treatment with the data at 6 month after the sitagliptin treatment started. Body weight, blood pressure, plasma glucose, hemoglobin A1c (HbA1c), serum low-density lipoprotein cholesterol (LDL-C), triglyceride (TG), high-density lipoprotein cholesterol (HDL-C), estimated glomerular filtration rate (e-GFR) in type 2 diabetic patients were measured almost at the same time points before and after 6-month-treatment with sitagliptin. Serum LDL-C levels were determined by direct measurement or the Friedewald’s formula.

### Statistical analyses

Differences in body weight, blood pressure, plasma glucose, HbA1c, serum lipids and e-GFR between before and after 6-month sitagliptin treatment were analyzed by the Paired t Test. Differences in the number of subjects with urinary glucose and protein before and after 6-month sitagliptin treatment were analyzed by the Pearson’s chi-squared test. We analyzed the correlation between change in body weight and body mass index (BMI) at baseline, and also the correlation between change in HbA1c and HbA1c levels at baseline by the Pearson's correlation test. P < 0.05 was considered to be statistical significant.

## Results

Body weight was significantly reduced after 6-month sitagliptin treatment by 0.8 kg (Table 3). HbA1c levels were also significantly decreased after the sitagliptin treatment by 0.6% (Table 3). However, there were no significant differences in systolic and diastolic blood pressure, plasma glucose, serum LDL-C, TG and HDL-C, e-GFR between before and after the sitagliptin treatment. Over half of subjects showed a reduction of body weight after the sitagliptin treatment, and HbA1c levels decreased in almost 70% of subjects (Table 4). Although a statistical significant difference was not obtained, switching from glinide to sitagliptin decreased body weight (n = 16; from 71.1 ± 11.6 kg to 69.0 ± 10.8 kg) and HbA1c (n = 17; from 7.4 ± 1.5% to 7.1 ± 1.6%), respectively. Almost 56% and 48% of subjects showed reduction of body weight and HbA1c by switching from glinide to sitagliptin.

**Table 3:** Changes in Clinical and Biochemical Data After 6 Month-Use of Sitagliptin

	Subjects studied (n)	Data after 6 month-use of sitagliptin	Changes compared with data before sitagliptin use	P value
Body weight (kg)	141	67.4 ± 15.8	-0.8 ± 3.7	< 0.001
Systolic blood pressure (mmHg)	169	125.2 ± 13.6	-1.5 ± 17.6	NS
Diastolic blood pressure (mmHg)	169	70.6 ± 11.0	+0.7 ± 12.4	NS
Plasma glucose (mg/dL)	195	175.7 ± 67.1	-9.8 ± 70.8	NS
Hemoglobin A1c (%)	169	7.5 ± 1.2	-0.6 ± 1.0	< 0.001
Serum LDL-C (mg/dL)	161	103.5 ± 25.6	-0.7 ± 27.6	NS
Serum TG (mg/dL)	161	169.4 ± 121.2	-7.3 ± 115.1	NS
Serum HDL-C (mg/dL)	161	49.8 ± 13.1	-0.9 ± 8.9	NS
e-GFR (mL/min./1.73m^2^)	199	75.9 ± 25.4	-1.5 ± 11.9	NS

Presented values indicate mean ± S.D. Statistical analyses have been done by Paired t Test. e-GFR, estimated glomerular filtration rate; HDL, high-density lipoprotein; LDL-C, low-density lipoprotein-cholesterol; NS, not statistically significant; TG, triglyceride.

**Table 4:** The Number and Percentage of Subjects Showed Increased, Unchanged, Decreased Values of Body Weight and Hemoglobin A1c After 6 Month-Use of Sitagliptin Compared with Values Before Sitagliptin Use

	Increased	Unchanged	Decreased
Body weight	46 (32.6%)	20 (14.2%)	75 (53.2%)
Hemoglobin A1c	44 (26.0%)	9 (5.3%)	116 (68.6%)

**Figure 1:**
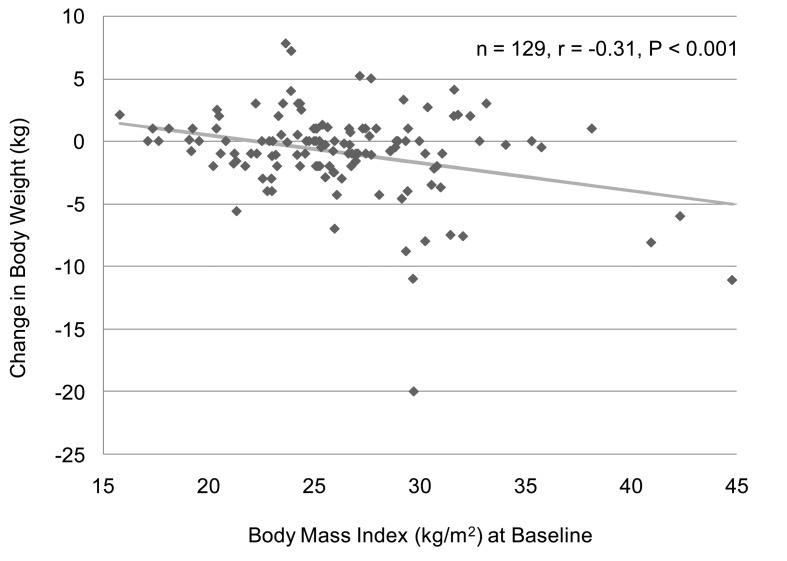
A correlation between body mass index at baseline and change in body weight after the 6-month sitagliptin treatment. A statistical analysis was performed by the Pearson's correlation test. r indicates correlation coefficient.

**Figure 2:**
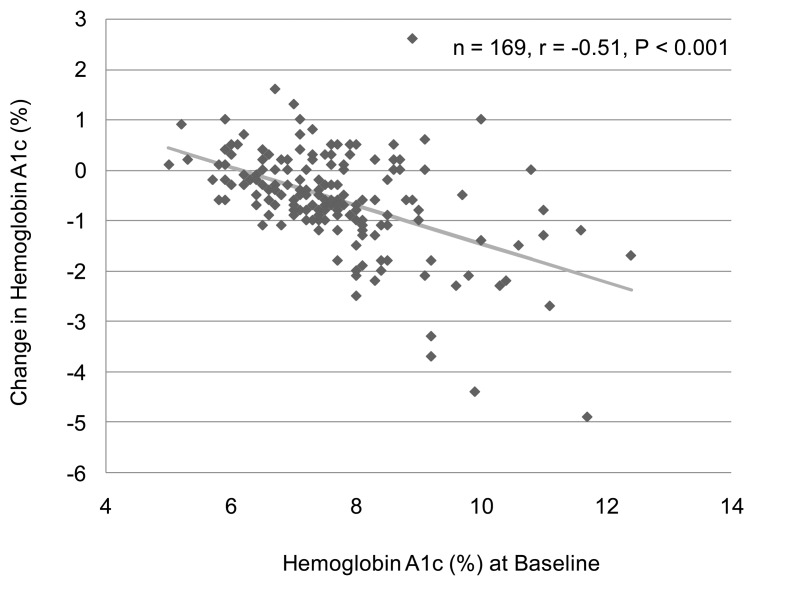
A correlation between hemoglobin A1c levels at baseline and change in hemoglobin A1c after the 6-month sitagliptin treatment. A statistical analysis was performed by the Pearson's correlation test. r indicates correlation coefficient.

We analyzed the correlation between change in body weight and BMI at baseline in 129 subjects who have data about BMI and body weight before and after the sitagliptin treatment. We found a significant and negative correlation between change in body weight and BMI at baseline (Fig. 1). However, there was no significant correlation between change in body weight and HbA1c at baseline (n = 126, r = 0.027, P = 0.76, the Pearson's correlation test). We also observed a significant and negative correlation between change in HbA1c and HbA1c levels at baseline (Fig. 2). We could not observe a significant correlation between change in HbA1c and BMI at baseline (n = 137, r = 0.051, P = 0.55, the Pearson's correlation test).

There were no significant differences in the number of patients with urinary protein between before and after the sitagliptin treatment (Table 5). However, the number of patients who showed the absence of urinary glucose was significantly increased after the sitagliptin treatment (Table 5).

**Table 5:** Changes in Urinary Glucose and Protein After 6 Month-Use of Sitagliptin

	Before sitagliptin use (n)	After sitagliptin use (n)	P value
Urinary glucose			
(-)	95	119	< 0.01
(+/-)	9	10	NS
(1+)	16	8	NS
(2+)	14	8	NS
(3+)	44	33	NS
Urinary protein			
(-)	104	97	NS
(+/-)	39	42	NS
(1+)	22	22	NS
(2+)	9	13	NS
(3+)	4	4	NS

Statistical analyses have been done by Pearson's chi-squared test. NS, not statistically significant.

## Discussion

We retrospectively studied effects of 6-month sitagliptin treatment on glucose and lipid metabolism, blood pressure, body weight and renal function in type 2 diabetic Japanese patients who had inadequate glycemic control on diet and exercise and by other oral antihyperglycemic agents by a chart-based analysis. In present study, HbA1c levels and body weight significantly decreased by 0.6% and by 0.8 kg, respectively, after 6 months after sitagliptin treatment started. However, we could not observe a significant influence of sitagliptin on blood pressure, serum lipids and e-GFR.

In a previous study in Colombia, patients (n = 455) treated with sitagliptin 100 mg monotherapy for 24 weeks have shown HbA1c change from baseline was -0.43%, and body weight change from baseline was -0.6 kg. HbA1c and BMI at baseline were 7.2 ± 0.5% and 30.7 ± 4.7 kg/m^2^, respectively [13]. In a study in Turkey (n = 28), sitagliptin 100 mg monotherapy for 12 weeks provided a reduction in HbA1c levels and body weight from baseline by 0.3% and 2.0 kg, respectively. In this study, HbA1c and BMI at baseline were 6.9 ± 0.7% and 31.6 ± 5.8 kg/m^2^, respectively [14]. The addition of sitagliptin (100 mg) to ongoing metformin therapy (n = 94) for 18 weeks decreased HbA1c and body weight by 0.73% and 0.4 kg, respectively, among patients in U.S.A. HbA1c and BMI at baseline were 7.8 ± 1.0% and 30.3 ± 4.7 kg/m^2^, respectively [15]. The addition of sitagliptin (100 mg) to ongoing pioglitazone therapy (n = 70) for 6 months decreased HbA1c and body weight by 0.5% and 0.6 kg, respectively, among patients in Italy. HbA1c and BMI at baseline were 8.5 ± 0.9 % and 27.9 ± 1.5 kg/m^2^, respectively [16].

In Japan, Nonaka K, et al (n = 75) demonstrated changes from baseline HbA1c and body weight with sitagliptin (100 mg) monotherapy for 12 weeks were -0.65% and -0.1 kg, respectively. HbA1c and BMI at baseline were 7.5 ± 0.9% and 25.2 ± 3.5 kg/m^2^, respectively [4]. Iwamoto Y, et al (n =155) reported that sitagliptin (50 mg) monotherapy for 12 weeks decreased HbA1c and body weight from baseline by 0.7% and 0.27 kg, respectively. HbA1c and BMI at baseline were 7.8 ± 0.9% and 24.5 ± 3.3 kg/m^2^, respectively [17]. Kashiwagi A, et al reported that changes in HbA1c and body weight at week 12 after the addition of sitagliptin (50 mg) to ongoing pioglitazone therapy compared with baseline was -0.4% and +0.4 kg, respectively (n = 66). HbA1c at baseline were 8.1 ± 0.9% [5]. HbA1c levels were decreased from baseline by 0.5% at 12 weeks after the addition of sitagliptin 50 mg to ongoing glimepiride, sulfonylurea, in Japanese type 2 diabetic patients (n = 71) [18]. HbA1c and BMI at baseline were 8.3 ± 0.8% and 24.6 ± 4.1 kg/m^2^, respectively. In this study, at week 52, the mean change form baseline in body weight was +0.28 kg.

Sitagliptin has been found to have better effect on HbA1c in Asian Indians and Koreans compared to Caucasians [11, 12, 15, 19, 20]. Daily 100 mg of sitagliptin decreased HbA1c by 0.3-0.73% in Caucasians [13-16], while daily 50 mg of sitaglitptin reduced HbA1c by 0.4-0.7% in Japanese [4, 5, 17, 18]. In our study, the mean ± SD of dose of sitagliptin which we used for the treatment was 48.6 ± 11.6 mg/day. Our study demonstrated daily dose under 50 mg of sitagliptin provided a great reduction in HbA1c (0.6%). Sitagliptin is likely to be effective to ameliorate HbA1c in also Japanese compared to Caucasians. Further, present study also demonstrated a significant and negative correlation between change in HbA1c and HbA1c levels at baseline, suggesting that sitagliptin is more effective to reduce HbA1c in patients in higher levels of HbA1c at baseline. Raz I, et al found that patients with higher baseline HbA1c (≥ 9%) experienced greater placebo-subtracted HbA1c reductions with sitagliptin (-1.20% for 100 mg and -1.04% for 200 mg) than those with HbA1c < 8% (-0.44% and -0.33%, respectively) or > or = 8% to 8.9% (-0.61% and -0.39%, respectively) after 18 weeks, which agrees with our observations [7].

Sitagliptin reduced body weight by 0.4 - 2.0 kg in Caucasians [13-16], while reduction of body weight by sitaglpitin was modest (-0.1 and -0.27 kg) in two Japanese studies which reported effects of sitagliptin monotherapy [4, 17], and sitagliptin slightly increased body weight (+0.4 and +0.28 kg) in two Japanese studies which investigated effects of sitagliptin add-on therapy [5, 18]. Sitagliptin is likely to be effective to reduce body weight in Caucasians compared to Japanese. However, in preset study, sitagliptin significantly decreased body weight by 0.8 kg. BMI (26.1 kg/m^2^) in our subjects is higher as compared with those in other Japanese studies [4, 5, 17, 18], which may lead to a significant reduction in body weight by sitagliptin, which is supported by a significant and negative correlation between change in body weight and BMI at baseline. Interestingly, sitagliptin is likely to be more effective to decrease body weight in patients with higher BMI at baseline.

Food intake, energy expenditure and urinary glucose excretion contribute to body weight change by the treatment using antihyperglycemic agents. Treatment of type 2 diabetic patients with oral antihyperglycemic agents improves glycemic control and results in the retention of calories that were excreted previously in the urine before the treatment [21]. Present study demonstrated that sitagliptin reduced urinary glucose excretion, suggesting that body weight reduction following the sitagliptin treatment was not due to increased urinary glucose excretion, may be due to a decrease in food intake or an increase in energy expenditure. Steven B, et al used a mathematical model to estimate the contribution of urinary glucose excretion to reported changes in body weight following oral antihyperglycemic agents therapy and found that body weight maintenance observed in response to DPP-4 inhibitors may result from an increase in satiety, energy expenditure, or both [22], supporting our observation and suggestion. Increases in GLP-1 concentration have been reported to increase satiety [23, 24], which could induce a decrease in food intake following DPP-4 inhibitors treatment. Further, fasting GLP-1 concentration has been reported to be positively correlated with resting energy expenditure [25]. Changes in satiety and energy expenditure may be sufficient to compensate for the positive energy balance due to reduced urinary glucose excretion.

In conclusion, our chart-based analysis of effects of 6-month sitagliptin treatment revealed that sitagliptin significantly reduce HbA1c, body weight and also urinary glucose excretion in Japanese type 2 diabetic patients. Further, present study showed a significant and negative correlation between change in body weight and body mass index at baseline, and also revealed a significant and negative correlation between change in HbA1c and HbA1c levels at baseline.

## References

[1] Drucker DJ, Nauck MA (2006). The incretin system: glucagon-like peptide-1 receptor agonists and dipeptidyl peptidase-4 inhibitors in type 2 diabetes. Lancet.

[2] Ahren B (1998). Glucagon-like peptide-1 (GLP-1): a gut hormone of potential interest in the treatment of diabetes. Bioessays.

[3] Ahren B (2010). Use of DPP-4 inhibitors in type 2 diabetes: focus on sitagliptin. Diabetes Metab Syndr Obes.

[4] Nonaka K, Kakikawa T, Sato A, Okuyama K, Fujimoto G, Kato N, Suzuki H (2008). Efficacy and safety of sitagliptin monotherapy in Japanese patients with type 2 diabetes. Diabetes Res Clin Pract.

[5] Kashiwagi A, Kadowaki T, Nonaka K, Taniguchi T, Mishii M, Ferreira JCA (2011). Sitagliptin added to treatment with ongoing pioglitazone for us to 52 weeks improves glycemic control in Japanese patients
with type 2 diabetes. J Diabetes Invest.

[6] Aschner P, Kipnes MS, Lunceford JK, Sanchez M, Mickel C, Williams-Herman DE (2006). Effect of the dipeptidyl peptidase-4 inhibitor sitagliptin as monotherapy on glycemic control in patients with type 2 diabetes. Diabetes Care.

[7] Raz I, Hanefeld M, Xu L, Caria C, Williams-Herman D, Khatami H (2006). Efficacy and safety of the dipeptidyl peptidase-4 inhibitor sitagliptin as monotherapy in patients with type 2 diabetes mellitus. Diabetologia.

[8] Goldstein BJ, Feinglos MN, Lunceford JK, Johnson J, Williams-Herman DE (2007). Effect of initial combination therapy with sitagliptin, a dipeptidyl peptidase-4 inhibitor, and metformin on glycemic control in patients with type 2 diabetes. Diabetes Care.

[9] Charbonnel B, Karasik A, Liu J, Wu M, Meininger G (2006). Efficacy and safety of the dipeptidyl peptidase-4 inhibitor sitagliptin added to ongoing metformin therapy in patients with type 2 diabetes inadequately controlled with metformin alone. Diabetes Care.

[10] Scott R, Wu M, Sanchez M, Stein P (2007). Efficacy and tolerability of the dipeptidyl peptidase-4 inhibitor sitagliptin as monotherapy over 12 weeks in patients with type 2 diabetes. Int J Clin Pract.

[11] Hermansen K, Kipnes M, Luo E, Fanurik D, Khatami H, Stein P (2007). Efficacy and safety of the dipeptidyl peptidase-4 inhibitor, sitagliptin, in patients with type 2 diabetes mellitus inadequately controlled on glimepiride alone or on glimepiride and metformin. Diabetes Obes Metab.

[12] Rosenstock J, Brazg R, Andryuk PJ, Lu K, Stein P (2006). Efficacy and safety of the dipeptidyl peptidase-4 inhibitor sitagliptin added to ongoing pioglitazone therapy in patients with type 2 diabetes: a 24-week, multicenter, randomized, double-blind, placebo-controlled, parallel-group study. Clin Ther.

[13] Aschner P, Katzeff HL, Guo H, Sunga S, Williams-Herman D, Kaufman KD, Goldstein BJ (2010). Efficacy and safety of monotherapy of sitagliptin compared with metformin in patients with type 2 diabetes. Diabetes Obes Metab.

[14] Oz O, Kiyici S, Ersoy C, Cander S, Yorulmaz H, Gul CB, Unal OK (2011). Effect of sitagliptin monotherapy on serum total ghrelin levels in people with type 2 diabetes. Diabetes Res Clin Pract.

[15] Scott R, Loeys T, Davies MJ, Engel SS (2008). Efficacy and safety of sitagliptin when added to ongoing metformin therapy in patients with type 2 diabetes. Diabetes Obes Metab.

[16] Derosa G, Maffioli P, Salvadeo SA, Ferrari I, Ragonesi PD, Querci F, Franzetti IG (2010). Effects of sitagliptin or metformin added to pioglitazone monotherapy in poorly controlled type 2 diabetes mellitus patients. Metabolism.

[17] Iwamoto Y, Tajima N, Kadowaki T, Nonaka K, Taniguchi T, Nishii M, Arjona Ferreira (2010). Efficacy and safety of sitagliptin monotherapy compared with voglibose in Japanese patients with type 2 diabetes: a randomized, double-blind trial. Diabetes Obes Metab.

[18] Tajima NA, Kadowaki T, Odawara M, Nishii M, Taniguchi T, Ferreira JCA (2011). Addition of sitagliptin to ongoing glimepiride therapy in Japanese patients with type 2 diabetes over 52 weeks leads to improved glycemic control. Diabetol Int.

[19] Mohan V, Yang W, Son HY, Xu L, Noble L, Langdon RB, Amatruda JM (2009). Efficacy and safety of sitagliptin in the treatment of patients with type 2 diabetes in China, India, and Korea. Diabetes Res Clin Pract.

[20] Williams-Herman D, Johnson J, Teng R, Luo E, Davies MJ, Kaufman KD, Goldstein BJ (2009). Efficacy and safety of initial combination therapy with sitagliptin and metformin in patients with type 2 diabetes: a 54-week study. Curr Med Res Opin.

[21] Geldermans CA, Terpstra J, Krans HM (1975). The effect of phenformin-HCl on patients with diabetes mellitus, studied under strict balance conditions. Diabetologia.

[22] Waters SB, Topp BG, Siler SQ, Alexander CM (2009). Treatment with sitagliptin or metformin does not increase body weight despite predicted reductions in urinary glucose excretion. J Diabetes Sci Technol.

[23] Flint A, Raben A, Astrup A, Holst JJ (1998). Glucagon-like peptide 1 promotes satiety and suppresses energy intake in humans. J Clin Invest.

[24] Flint A, Raben A, Ersboll AK, Holst JJ, Astrup A (2001). The effect of physiological levels of glucagon-like peptide-1 on appetite, gastric emptying, energy and substrate metabolism in obesity. Int J Obes Relat Metab Disord.

[25] Pannacciulli N, Bunt JC, Koska J, Bogardus C, Krakoff J (2006). Higher fasting plasma concentrations of glucagon-like peptide 1 are associated with higher resting energy expenditure and fat oxidation rates in humans. Am J Clin Nutr.

